# Tackling reference bias in genotyping by using founder sequences with PanVC 3

**DOI:** 10.1093/bioadv/vbae027

**Published:** 2024-03-04

**Authors:** Tuukka Norri, Veli Mäkinen

**Affiliations:** Applied Tumor Genomics Research Program, Faculty of Medicine, University of Helsinki, FI-00014 Helsinki, Finland; Department of Computer Science, University of Helsinki, FI-00014 Helsinki, Finland

## Abstract

**Summary:**

Overcoming reference bias and calling insertions and deletions are major challenges in genotyping. We present *PanVC 3*, a set of software that can be utilized as part of various variant calling workflows. We show that, by incorporating known genetic variants to a set of founder sequences to which reads are aligned, reference bias is reduced and precision of calling insertions and deletions is improved.

**Availability and implementation:**

PanVC 3 and its source code are freely available at https://github.com/tsnorri/panvc3 and at https://anaconda.org/tsnorri/panvc3 under the MIT licence. The experiment scripts are available at https://github.com/algbio/panvc3-experiments.

## 1 Introduction

Recently genotyping methods have been developed to overcome reference bias towards the variation chosen for a reference genome used as part of the process (Popejoy and Fullerton 2016). The methods typically represent some known variation as a graph, to which nucleotide sequence reads are then aligned (e.g. Garrison *et al.* 2018, Pritt *et al.* 2018, Sirén *et al.* 2021). While variant calling tools that utilize a single reference sequence have been improved over a long time (e.g. McKenna *et al.* 2010, DePristo *et al.* 2011, Van der Auwera *et al.* 2013, Poplin *et al.* 2018, Van der Auwera and O’Connor 2020), ones that utilize a graph are relatively new.

In this article, we refine an existing pangenomic variant calling method, PanVC+founders (Norri *et al.* 2021), that is based on exploiting few founder sequences to represent the variation. In the original approach, these founder sequences are used in place of a standard linear reference genome in read alignment. Then these read alignments are used for creating an *ad hoc* reference as a prediction of the donor genome accompanied with a set of heterozygous variants present in the input. Novel variants are then called using re-alignment of reads to the *ad hoc* reference. As the last step, the resulting combined set of variants is projected to the reference genome. This last step has turned out to have some caveats: The projection step loses information such as mapping quality, and many downstream analysis tools need read alignments as the input instead of the variants. To overcome these caveats, we redesigned the approach to skip the creation of the *ad hoc* reference. Instead, we project the read alignments directly to the standard reference and adjust mapping quality values accordingly. With these modifications, it is easier to use the output of our software toolset in downstream analysis while also improving the results of genotyping insertions and deletions and reducing bias towards the reference.

**Figure 1.**
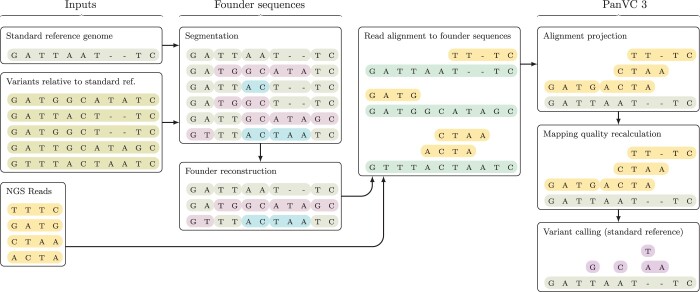
Example genotyping workflow that utilizes PanVC 3. The inputs are shown on the left. The alignments have been chosen for demonstration purposes and are not necessarily optimal.

Our resulting toolset is scalable and can be seamlessly integrated into various genotyping workflows, in particular into ones that utilize a linear reference genome and make use of short reads. Such use case is illustrated in Fig. 1 and the differences to the previous version of PanVC are shown in Supplementary Fig. S1. In the following, we describe the different steps of the refined approach in detail, focusing on the novel parts. Then we proceed with experiments and conclude with some remarks on future directions.

## 2 Algorithm and implementation

As mentioned, our toolset can be utilized to align reads to a set of reference sequences, after which the alignments can be projected to a standard reference sequence for downstream analysis (see *alignment projection* and *variant calling* in Fig. 1). To this end, a multiple sequence alignment of the reference sequences is needed. The alignment need not be optimal, though; a *reference-guided* multiple sequence alignment that is consistent with the given pairwise alignments suffices (Norri *et al.* 2021).

### 2.1 Founder sequences are utilized to reduce the number of indexed sequences

To make read alignment to multiple sequences scalable, we produce a number of *founder sequences* (Ukkonen 2002) from known variants instead of utilizing e.g. the complete predicted haplotype sequences of some group of individuals. Founder sequences have the property that each input haplotype can be read as a recombination of founders. For generating the founder sequences, we use scalable methods that produce a small number of founders while minimizing the number of recombinations i.e. possible discontinuation positions (Norri *et al.* 2021). This process is illustrated in Fig. 1 (*segmentation* and *founder reconstruction*).

### 2.2 Alignments are projected to standard reference

Next, the unaligned founder sequences and the reads are used as an input to a chosen read aligner (see *read alignment to founder sequences* in Fig. 1). To make alignment projection possible in a reasonable time, a simple co-ordinate projection data structure is generated from the multiple sequence alignment of the founder sequences. Building the data structure and applying the co-ordinate transformation are described in Supplementary Section S1.

Typically read aligners report the corresponding parts of an aligned read and the reference sequence as a run-length encoded sequence of *edit operations* that include match, mismatch, and insertion and deletion in the read. As part of alignment projection, the edit operations are rewritten with the algorithm described in Supplementary Section S2. The operations can be rewritten in linear time with respect to their count, not taking into account the time required to realign parts of the corresponding read.

### 2.3 Mapping qualities are recalculated

Read aligners also report a *mapping quality* for each alignment, which variant callers may then utilize to assess the alignments. The mapping quality is defined as $-10{\;\text{log}\;}_{10}p$ where *p* is an estimate given by the read aligner that the read in question has been incorrectly *aligned* (The SAM/BAM Format Specification Working Group 2022). In practice, read aligners may produce the estimate based on the properties of the alignment.

One such approach, implemented in Bowtie 2 (Langmead and Salzberg 2012), is based on first calculating an *alignment score* for each aligned read. This is done by penalizing mismatches, insertions, and deletions in the alignment. Base-quality scores can also be taken into account. An estimated mapping quality is then determined from the scores of the alignment in question and the next best alignment if one exists. A large difference of the alignment scores yields a high mapping quality.

Since matching parts of founder sequences can be identical to each other, one read can have multiple equivalently scored alignments with the same projected leftmost co-ordinate. As the read aligner does not apply co-ordinate projection, it can assign very low mapping qualities to the alignments. To solve the issue, we partitioned the set of alignments of each read by the projected leftmost co-ordinate. We then used the alignment scores of the representative elements of the partitions as inputs to Bowtie 2’s mapping quality estimation algorithm, which we reimplemented for this purpose (see *mapping quality recalculation* in Fig. 1).

Some other tools have functionality for considering alternative reference sequences, which can be utilized with founder sequences. For instance, BWA (Li and Durbin 2009) has the necessary functionality but, based on our tests, it does not report the alignment scores of the alignments to the alternative sequences. To process such alignments for use with PanVC 3, we also implemented Bowtie 2’s alignment scoring algorithm.

## 3 Methods

### 3.1 Reference bias experiment

To evaluate our approach, we measured the reference bias of the projected alignments with an experiment similar to the one used to evaluate FORGe (Pritt *et al.* 2018). We calculated the bias from simulated reads of NA12878’s chromosome 1 generated with Mason (Holtgrewe 2010). As the truth set, we used heterozygous variants from the Genome-in-a-Bottle v4.2.1 small variant call set (Wagner *et al.* 2022) relative to the GRCh37 reference assembly.

We aligned the reads and produced a pileup for each variant site. Alignments were considered only if the aligned read fully enclosed the variant site and contained at least one match or mismatch edit operation after it, and the coverage was at least 20. From the pileup, we counted the aligned reads that supported the reference allele and the correct alternative allele and calculated their ratio (i.e. $\frac{R}{R+A}$ where *R* and *A* are the counts of reads that support, respectively, the reference allele and the correct alternative allele). Since an equal amount of simulated reads was generated from each haplotype, the ratios should ideally be very close to 0.5.

**Figure 2.**
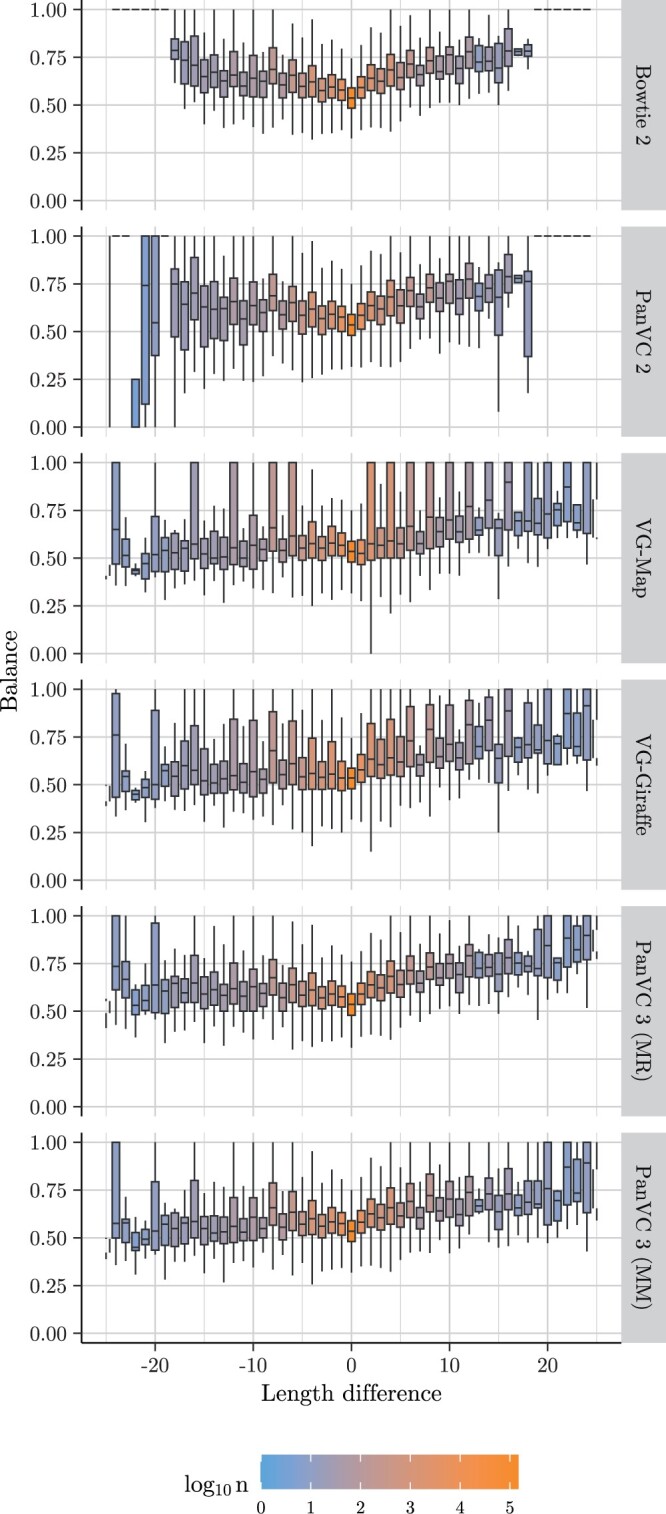
Proportions $\frac{R}{R+A}$ (‘balances’) by the difference of the lengths of the alternative allele and the reference allele in the reference bias experiment. The proportion is shown on the *Y* axis, the length difference on the *X* axis, and the value *n* shown with the fill colour indicates the number of alignments in each equivalence class. The median balance is shown with a horizontal line inside each box, and the lower and upper hinges correspond to the first and third quartiles. The whiskers show the smallest and the largest value such that the difference to respectively the first and third quartiles is at most 1.5 times the distance between those quartiles; more distant values (outliers) have been omitted. The tested workflow is shown on the right of each row. MM indicates that the alignments were filtered by maximizing the mapping quality while MR indicates that no filtering was done.

In addition to the current version of PanVC, reference bias was calculated from alignments to the hs37d5 reference with Bowtie 2 (Langmead and Salzberg 2012) and with VG-MAP (Garrison *et al.* 2018) as well as Giraffe (Sirén *et al.* 2021). The previous version of PanVC was also tested by projecting the benchmark variant co-ordinates to those of the *ad hoc* reference sequence. In the case of both versions of PanVC, Bowtie 2 was used as the read aligner. Details of the experiment are provided in Supplementary Section S3.1. The results are shown in Fig. 2 and Supplementary Fig. S2.

In addition to the previously mentioned read aligners, we attempted to test Reference Flow (Chen *et al.* 2020), which also utilizes alignment projection. However, despite our best efforts, we were unable to produce meaningful results with it. The problems may have had to do with the fact that the software is still being refined. (The relevant issue is located at https://github.com/langmead-lab/reference_flow/issues/3).

**Figure 3.**
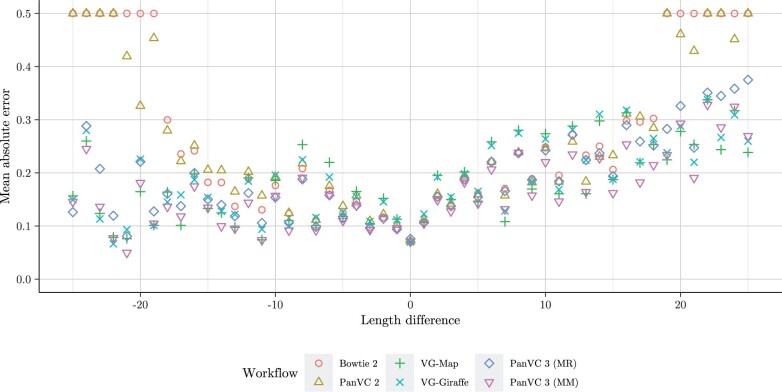
Mean absolute error values of the ratios calculated by the difference of the lengths of the alternative allele and the reference allele in the reference bias experiment. The error is shown on the *Y* axis and the length difference on the *X* axis. The error values have been calculated with the formula $\frac{1}{n}\sum_{1}^{n}|x_{i}-0.5|$ where $x_{i}$ are the proportions $\frac{R}{R+A}$ and *R* and *A* are the counts of reads that support, respectively, the reference allele and the correct alternative allele. The point shape and colour correspond to the tested workflow. MM indicates that the alignments were filtered by maximizing the mapping quality while MR indicates that no filtering was done.

To assess the results, we calculated the mean absolute errors of the aforementioned ratios with respect to the value 0.5 i.e. $\frac{1}{n}\sum_{1}^{n}|x_{i}-0.5|$ where $x_{i}$ are the ratios. We also calculated the overall mean absolute error for each alignment workflow. The results are shown in Fig. 3 and Supplementary Table S2.

#### 3.1.1 Alignment precision and recall

**Figure 4.**
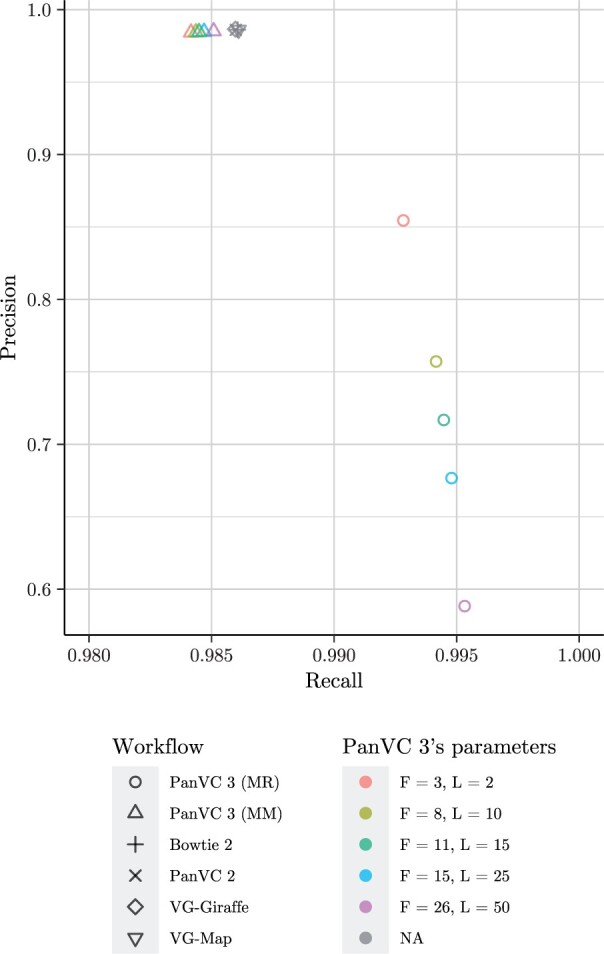
Alignment precision and recall in the reference bias experiment. The point shape corresponds to the workflow and the colour to PanVC 3’s parameters. *F* indicates the founder sequence count including the reference sequence, *L* is the minimum distance between subgraphs, and MM indicates that the alignments were filtered by maximizing the mapping quality while MR indicates that no filtering was done.

To evaluate the alignments, we also compared the aligned position of each read to the correct position as reported by Mason. We considered an alignment a true positive if its distance from the correct position was at most five bases. If the read was aligned to some other position, we considered it a false negative. Finally, if a read was reported as not aligned or was missing from the alignment results, we considered it a false negative. Consequently, the sum of the true positives and false negatives equalled the number of the reads. From these counts, we calculated precision and recall. The results are shown in Fig. 4.

### 3.2 Structural variant calling experiment

We tested five workflows for calling structural variants from Human Pangenome Reference Consortium’s (Zook *et al.* 2016) sequencing data for NA24385. In a baseline workflow, the reads were aligned to hs37d5 (The 1000 Genomes Project Consortium *et al.* 2015). The second workflow used PanVC 3, as shown in Fig. 1. In this case, the reads were aligned to a set of sequences that consisted of hs37d5 and a varying number of founder sequences of chromosome 1. The founder sequences were generated from the phase 3 variant data from the 1000 Genomes project (The 1000 Genomes Project Consortium *et al.* 2015) which did not include NA24385 or their close relatives. The third tested workflow was similar to the second one, except that only the alignment with the best mapping quality for each read was retained. The fourth and fifth tested workflows utilized, respectively, VG-MAP (Garrison *et al.* 2018) and Giraffe (Sirén *et al.* 2021) with the same variant data. PanVC 2 was not included in this experiment because its variant projection tool has only been tested with GATK (Van der Auwera and O’Connor 2020) and BCFtools (Danecek *et al.* 2021) and does not handle structural variants correctly. Similarly to the reference bias experiment, we also tried to test Reference Flow (Chen *et al.* 2020) but did not get any meaningful results.

**Figure 5.**
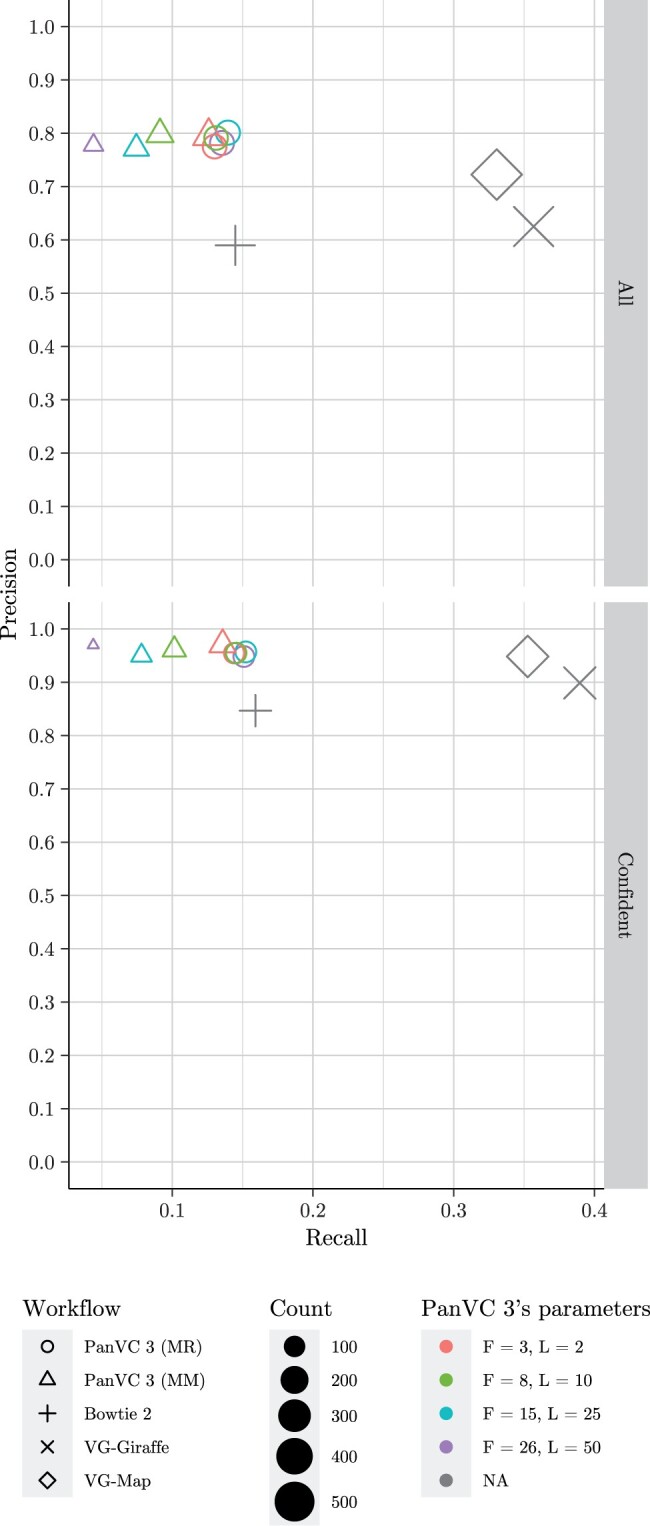
Precision and recall as reported by Truvari and the called variant counts in the structural variant calling experiment for all (top graph) and confident regions (bottom graph) of chromosome 1, considering only variants reported by Manta that passed all filters. The point shape corresponds to the workflow and the colour to PanVC 3’s parameters. *F* indicates the founder sequence count including the reference sequence, *L* is the minimum distance between subgraphs, and MM indicates that the alignments were filtered by maximizing the mapping quality while MR indicates that no filtering was done.

In the baseline and the PanVC 3 workflows, Bowtie 2 (Langmead and Salzberg 2012) was used as the read aligner. With each set of alignments, Manta (Chen *et al.* 2015) was used for variant calling. The variants were then evaluated with Truvari (English *et al.* 2022) against the Genome-in-a-Bottle v0.6 Tier 1 structural variant benchmark set (Zook *et al.* 2020). Details of the experiment are provided in Supplementary Section S3.2. The results are shown in Fig. 5 and Supplementary Tables S3 and S4.

### 3.3 Take-one-out experiment with a human chromosome

Finally, we repeated one of the experiments used to test the previous version of PanVC. Sequencing data for NA12878 from the Illumina Platinum Genomes project (Eberle *et al.* 2017) were used for testing. To save computing resources, we sampled the reads to lower the coverage to approximately half of the original.

Similarly to the other experiments, we also tested three other read aligners, namely Bowtie 2 (Langmead and Salzberg 2012), VG-MAP (Garrison *et al.* 2018), and Giraffe (Sirén *et al.* 2021). In the case of VG-MAP and Giraffe, the phase 3 variant data relative to the hs37d5 reference from the 1000 Genomes Project (The 1000 Genomes Project Consortium *et al.* 2015) after removing NA12878 and their close relatives from the set of samples were used as indexing input. In the case of PanVC 3, a varying number of founder sequences were generated from the same data. In the case of PanVC 2, a set of three founder sequences generated from said variant data with the maximum distance between subgraphs set to two was used as indexing input. These parameters were chosen since they yielded good results in our earlier experiments with PanVC 2.

**Figure 6.**
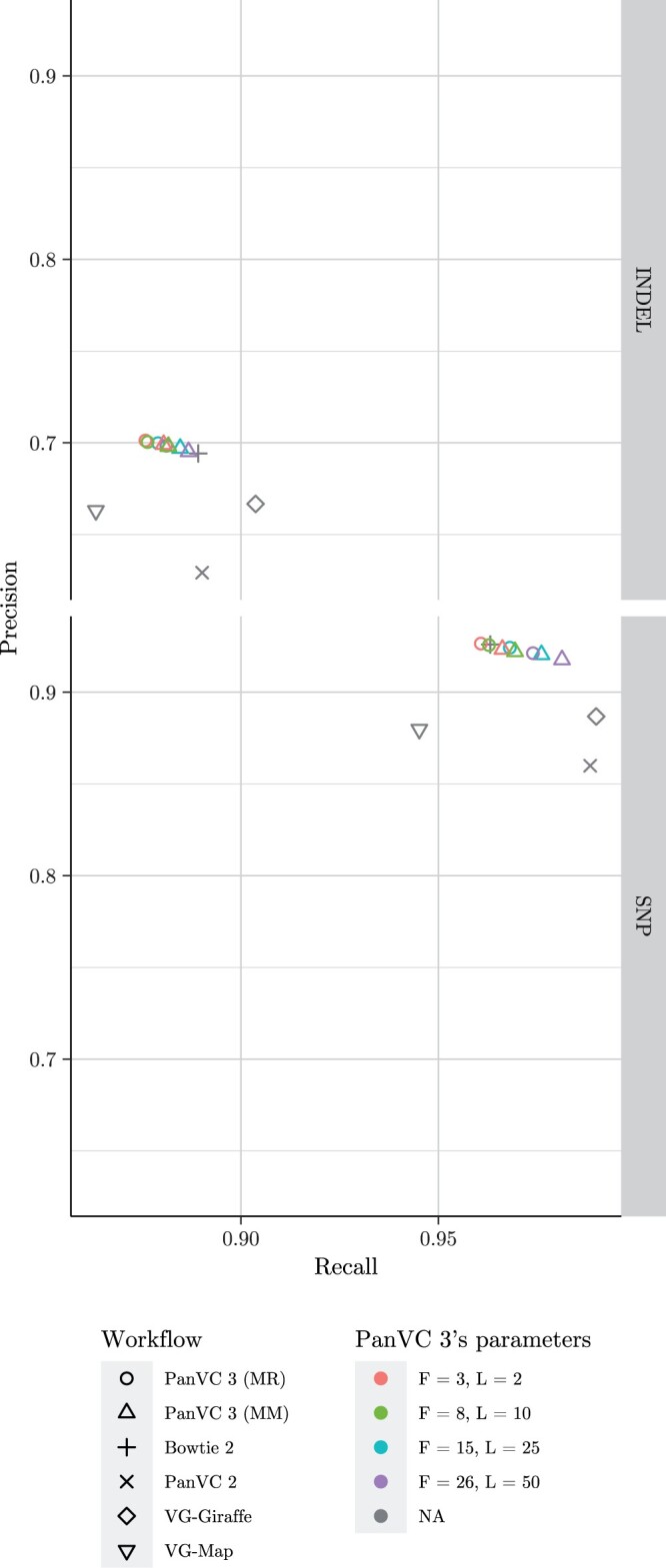
Precision and recall as reported by hap.py for short insertions and deletions (top graph) and single nucleotide polymorphisms (bottom graph) in the take-one-out experiment with a human chromosome, considering all regions of chromosome 1. The point shape corresponds to the workflow and the colour to PanVC 3’s parameters. *F* indicates the founder sequence count including the reference sequence, *L* is the minimum distance between subgraphs, and MM indicates that the alignments were filtered by maximizing the mapping quality while MR indicates that no filtering was done.

We used GATK (Van der Auwera and O’Connor 2020) for variant calling and evaluated the results with hap.py from Illumina’s Haplotype Comparison Tools (https://github.com/Illumina/hap.py). We used variant calls from the Genome-in-a-Bottle v4.2.1 small variant call set (Wagner *et al.* 2022) as a benchmark. Results of the experiment are shown in Fig. 6 and Supplementary Fig. S5. Details of the experiment are provided in Supplementary Section S3.3.

## 4 Results

The results of the reference bias experiment indicate that there was a notable decrease in reference bias in PanVC 3 compared to the baseline workflow, as well to the previous version of PanVC for alignments to the longer-inspected insertions and deletions (ca. 25 nucleotides or more). Furthermore, the mean absolute error values were the smallest in the case of PanVC 3 with mapping quality maximization both in the overall case as well as in the case of 30 of the 51 examined length differences.

The results on alignment precision and recall indicate that utilizing founder sequences can increase recall by over one percent point. On the other hand, postprocessing the alignments by maximizing the mapping quality caused the recall to drop below that of the other tested workflows, which produced very similar results compared to each other.

Considering the other two experiments, using PanVC 3 the SNP genotyping recall and the structural variant genotyping precision were clearly improved upon those of the baseline. The latter difference was particularly clear when all regions of chromosome 1 were considered. On the other hand, in both experiments, using Giraffe resulted in better recall. However, in the structural variant calling experiment considering all regions and in the take-one-out experiment, better recall was achieved with PanVC 3 even compared to Giraffe.

In the take-one-out experiment, PanVC 2 had slightly better recall in genotyping SNPs and short insertions and deletions. However, PanVC 3 had much better precision.

Postprocessing PanVC 3’s alignments by maximizing the mapping quality of each read produced somewhat mixed results: in the take-one-out experiment, applying this step improved precision of genotyping both SNPs and short insertions and deletions, while in the structural variant calling experiment including the alternative alignments in Manta’s input made the results better. Postprocessing the alignments in the reference bias resulted in a lower recall than in the case of the other tested workflows, while expectedly the recall increased by omitting the step. The results indicate that considering other options for postprocessing the alignments could be worthwhile.

In the take-one-out experiment, increasing the number of founder sequences and maximizing the mapping quality of the alignments improved recall, while the effect seemed to be the opposite in the structural variant calling experiment. On the other hand, using a moderate number of founder sequences without applying the postprocessing step yielded the best results in terms of precision. Different variant callers were used in the experiments, which likely contributed to the dissimilarity.

In the reference bias experiment, the balance values of the even allele length differences were quite dissimilar to those of the odd ones in the case of many of the tested workflows. Similarly, the calculated mean absolute error values were higher for the even allele length differences. Unfortunately, we do not have an explanation for this.

## 5 Discussion

We developed and tested an approach of using multiple reference sequences and projecting the alignments to a common reference sequence in a genotyping workflow based on short reads. Our experiments with simulated reads indicate that our approach reduces bias towards the variation chosen for the reference genome and works well in comparison to both conventional and graph-based workflows. Furthermore, based on our experiments with biological data, we were able to improve precision in calling long insertions and deletions over a workflow that utilizes only one reference sequence. We note that our workflow was relatively simple, and our tools can be integrated into different, more complex workflows that are based on short reads.

One option for further development is to test the workflow with long reads. A potential problem stems from representing a given subsequence of a genome that spans the length of a long read with a small number of founder sequences. In such cases, the representation could have a large number of breakpoints which could affect aligning reads correctly.

Further considerations are recounted in Supplementary Section S4.

## Supplementary Material

vbae027_Supplementary_Data

## Data Availability

PanVC 3 is available in Github at https://github.com/tsnorri/panvc3 and in Anaconda at https://anaconda.org/tsnorri/panvc3. The data underlying this article are available in GitHub at https://github.com/algbio/panvc3-experiments.
