# Supplementary material for: Tackling reference bias in genotyping by using founder sequences with PanVC 3
Source: Bioinform Adv. 2024 Mar 4;4(1):vbae027. doi: 10.1093/bioadv/vbae027 (PMC10924279; doi:10.1093/bioadv/vbae027)
Supplement: vbae027_Supplementary_Data [file vbae027_supplementary_data.pdf]

Supplement:
Tackling Reference Bias in Genotyping by Using Founder Sequences with PanVC 3

Tuukka Norri and Veli Mäkinen

February 2024

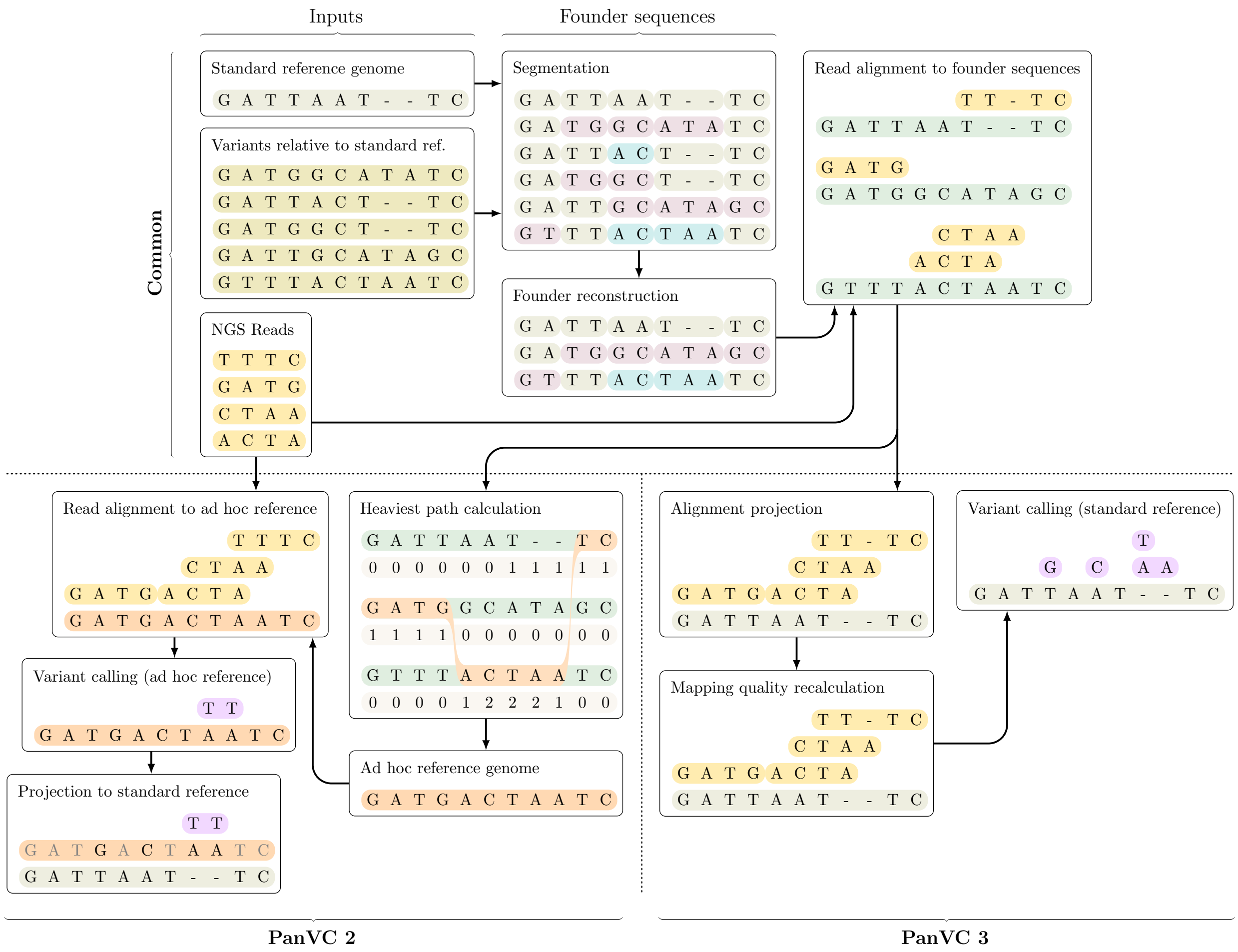

Supplemental Figure 1: Example genotyping workflows that utilise the new and, for comparison, the previous version of PanVC (respectively "PanVC 3" and "PanVC 2"). The inputs are shown on top left, the common phases on the top, and the phases specific to either version respectively at the bottom right and left. The alignments have been chosen for demonstration purposes and are not necessarily optimal. A key difference is that due to projecting the alignments to the reference sequence co-ordinates, only one alignment phase is necessary in the new version, whereas in PanVC 2 the reads were realigned to the *ad hoc reference* constructed using the initial alignments. Due to the differences in design, the workflows can predict different variants.

1 Co-ordinate Transformation Data Structure

To project co-ordinates between aligned sequences, the following simple data structure is used. For each sequence of n characters, a bit vector B of n elements is built. The element $B[i]$ is set to 0 if the i-th character of the sequence is a gap, and otherwise to 1.

Suppose the co-ordinate k of some *source* sequence is to be projected to a *destination* sequence that has been aligned to the source. Also, suppose the corresponding bit vectors are respectively called B_S and B_D. We first retrieve the position of the k-th one in B_S using the *select* operation, thus converting the co-ordinate to that of the multiple sequence alignment. To retrieve the position in the destination sequence, we count the ones in B_D up to that position with the *rank* operation. Using suitable data structures, this can be done in constant time after preprocessing the bit vectors in linear time (Babenko *et al.*, 2015). To save space, a compressed representation of the bit vectors can be used (Navarro and Providel, 2012).

2 Alignment Projection Algorithm

The run-length encoded sequences of edit operations that read aligners typically use to report the corresponding parts of an aligned read and the reference sequence are known as *CIGAR strings* (The SAM/BAM Format Specification Working Group, 2022). To rewrite the edit operations, the sequence of operations is processed from beginning to end. Insertions in the read are initially added to the rewritten sequence of edit operations without modifications. Other types of operations are said to *consume reference*, that is, indicate that the alignment steps along the source sequence. To handle such an operation, the projected co-ordinate of the current source sequence character is first compared to that of the previous character. If there are additional characters in the destination sequence between these positions, an equivalent number of deletion operations is added to the alignment. Next, the alignment operation is rewritten according to the rules listed in Supplemental Table 1. After all the operations have been rewritten, the resulting sequence of edit operations is checked for maximal runs of insertion and deletion operations that contain at least one of each type, and the corresponding runs of characters in the read and the destination sequence are identified. Finally, the aforementioned parts of the read and the destination sequence are realigned and the rewritten sequence of edit operations is modified accordingly.

Similarly to projecting the co-ordinates, the rank operation can be used to determine the number of characters in the destination sequence between a pair of multiple sequence alignment co-ordinates in constant time. Hence, the linear time complexity with respect to the number of operations is achieved, not taking into account the time required to realign parts of the corresponding read.

3 Details of the Experiments and Additional Results

3.1 Reference Bias Experiment

We produced simulated reads from NA12878's chromosome 1. To this end, we generated predicted haplotype sequences from the heterozygous variants in the Genome-in-a-Bottle project v4.2.1 small variant call set (Wagner *et al.*, 2022) and the hs37d5 reference sequence and treated the variants as phased. We then used Mason (Holtgrewe, 2010) to generate 30 million paired-ended reads for each haplotype, resulting in a mean coverage of 24.3 per haplotype with 90.38 % of all bases covered. The reads were then aligned with each of the tested workflows.

In case of Reference Flow, VG-MAP and Giraffe, the phase 3 variant data for chromosome 1 from the 1000 Genomes project (The 1000 Genomes Project Consortium *et al.*, 2015) was used as indexing input, having first removed NA12878. In the case of PanVC 2, a set of 3 founder sequences generated from said variant data with the maximum distance between subgraphs set to 2 was used as indexing input. These parameters were chosen since they yielded good results in our earlier experiments with PanVC 2.

In the case of PanVC 3, a set of 11 founder sequences including the reference sequence was used. The founder sequences were generated from the aforementioned variant data. A post-processing step was also

Supplemental Table 1: Rewriting rules for the edit operations in alignment projection. The middle columns ("Destination has gap" and "Match") respectively indicate whether the destination sequence has a gap character, and whether the character in the read matches that in the destination sequence.

Original op.	Destination has gap	Match	Rewritten op.
Match or mismatch	no	yes	Match
Match or mismatch	no	no	Mismatch
Deletion	no		Deletion
Match or mismatch	yes		Insertion
Deletion	yes		no-op

Supplemental Table 2: Overall mean absolute error values of the ratios calculated in the reference bias experiment such that the lengths of the reference allele and the alternative allele differ by at most 25 nucleotides. The values have been calculated with the formula $\frac{1}{n}\sum_{1}^{n}|x_i - 0.5|$ where x_i are the proportions $\frac{R}{R+A}$ and R and A are the counts of reads that support respectively the reference allele and the correct alternative allele. MM indicates that the alignments were filtered by maximising the mapping quality while MR indicates that no filtering was done.

Workflow	Mean absolute error
Bowtie 2	0.0778
PanVC 2	0.0785
VG-MAP	0.0782
VG-Giraffe	0.0783
PanVC 3 (MR)	0.0814
PanVC 3 (MM)	**0.0763**

tested where the mapping qualities were recalculated and only the alignment with the best mapping quality for each read was kept.

3.1.1 Computational requirements

We benchmarked the indexing and alignment steps of the workflows that utilised Bowtie 2, VG-MAP, VG-Giraffe, PanVC 2, and PanVC 3 with Snakemake's benchmark feature. To evaluate memory consumption, we measured the maximum resident set size for each step. Additionally, we measured the wall clock time to run each workflow. The latter benchmark can be considered approximate, as the server used to test the workflows is shared and the I/O operations are taken into account.

The machine used ran Ubuntu Linux version 14.04.5 LTS and had 172 GB of RAM and 40 Intel Xeon (Skylake, IBRS) processors, 2.4 GHz each. Bowtie 2, vg, and Samtools were run parallelised; Bowtie 2 and vg used 32 threads. The results are shown in Supplemental Figures 3 and 4.

The results indicate that generating the founder sequences does not considerably increase the time required for generating the index with the inputs of this experiment. On the other hand, projecting the alignments does increase the time needed for aligning the reads substantially. The reason for this may be that the projection tool is relatively new and has not yet been parallelised. On the other hand, projecting the alignments as well as recalculating and maximising the mapping quality require very little memory. We also note that with the current version, parallelisation of the alignment projection could be achieved by splitting the input (e.g. by chromosome or even arbitrarily).

3.2 Structural Variant Calling Experiment

Sequencing data for NA24385 from the Human Pangenome Reference Consortium (Zook *et al.*, 2016) were used for testing structural variant calling accuracy. The data consisted of paired-end 150 bp Illumina reads with approximately 450 bp fragment size, downsampled to approximately 30x coverage. The reads were aligned with Bowtie 2 (Langmead and Salzberg, 2012) and Manta (Chen *et al.*, 2015) was used for variant calling. Only variants in chromosome 1 were considered; to this end, Manta's `--region` option was used to limit variant calling to the chromosome in question. Finally, the variants were evaluated with Truvari (English *et al.*, 2022) against the Genome-in-a-Bottle v0.6 Tier 1 structural variant benchmark set (Zook *et al.*, 2020), considering only the regions listed with the data set that were located in chromosome 1, and variants with the FILTER field set to PASS.

For Reference Flow, VG-MAP and Giraffe, the phase 3 variant data for chromosome 1 from the 1000 Genomes project (The 1000 Genomes Project Consortium *et al.*, 2015) was used as indexing input. In the case of PanVC 3, the founder sequences were generated from the same variant data. In the baseline workflow,

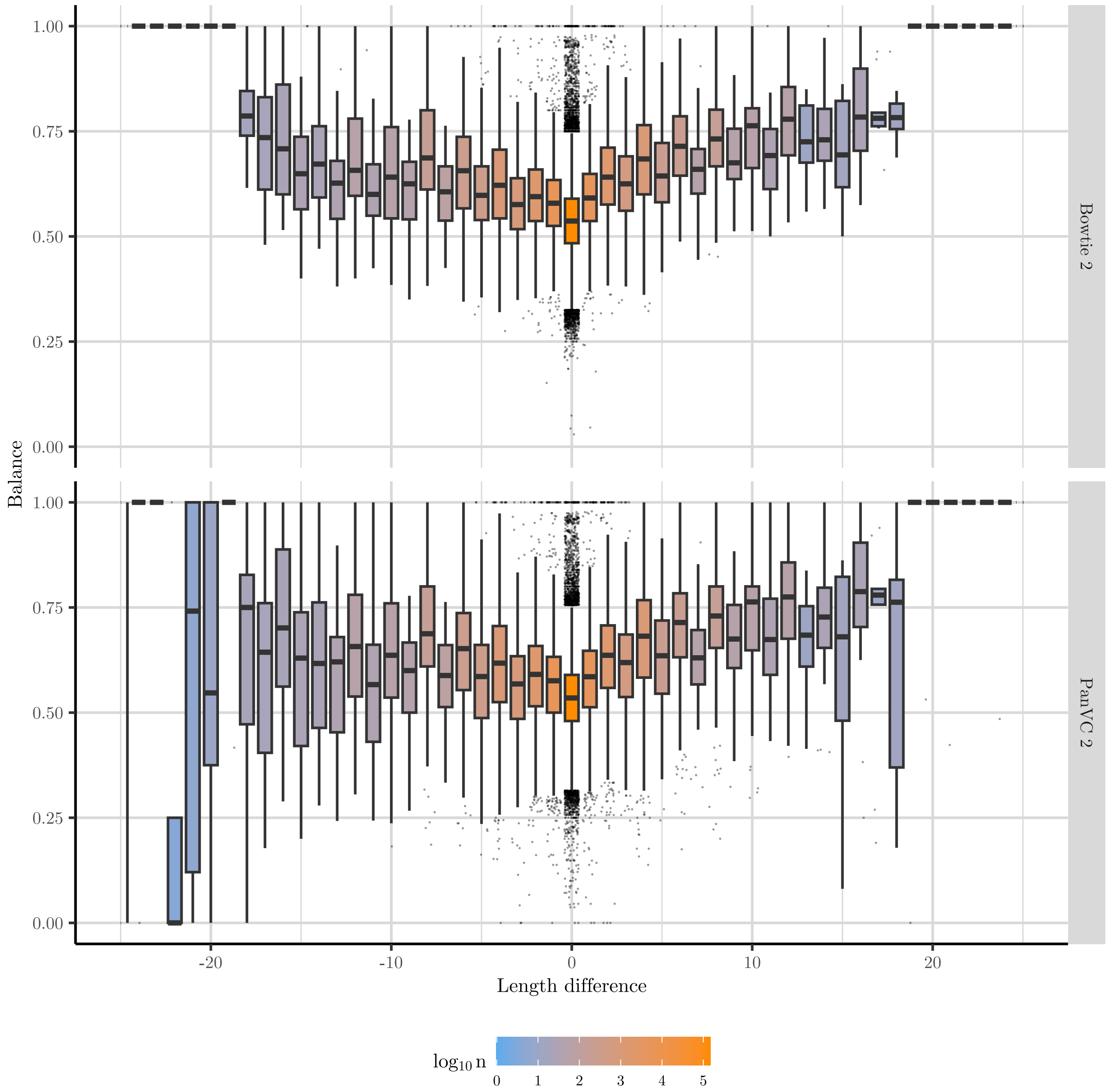

(a) Results for the Bowtie 2 and PanVC 2 workflows.

Supplemental Figure 2: Proportions $\frac{R}{R+A}$ ("balances") by the difference of the lengths of the alternative allele and the reference allele in the reference bias experiment. The proportion is shown on the Y axis, the length difference on the X axis, and the value n shown with the fill colour indicates the number of alignments in each equivalence class. The median balance is shown with a horizontal line inside each box, and the lower and upper hinges correspond to the first and third quartiles. The whiskers show the smallest and the largest value such that the difference to respectively the first and the third quartile is at most 1.5 times the distance between those quartiles; more distant values (outliers) have been plotted individually with dots. The tested workflow is shown on the right of each row.

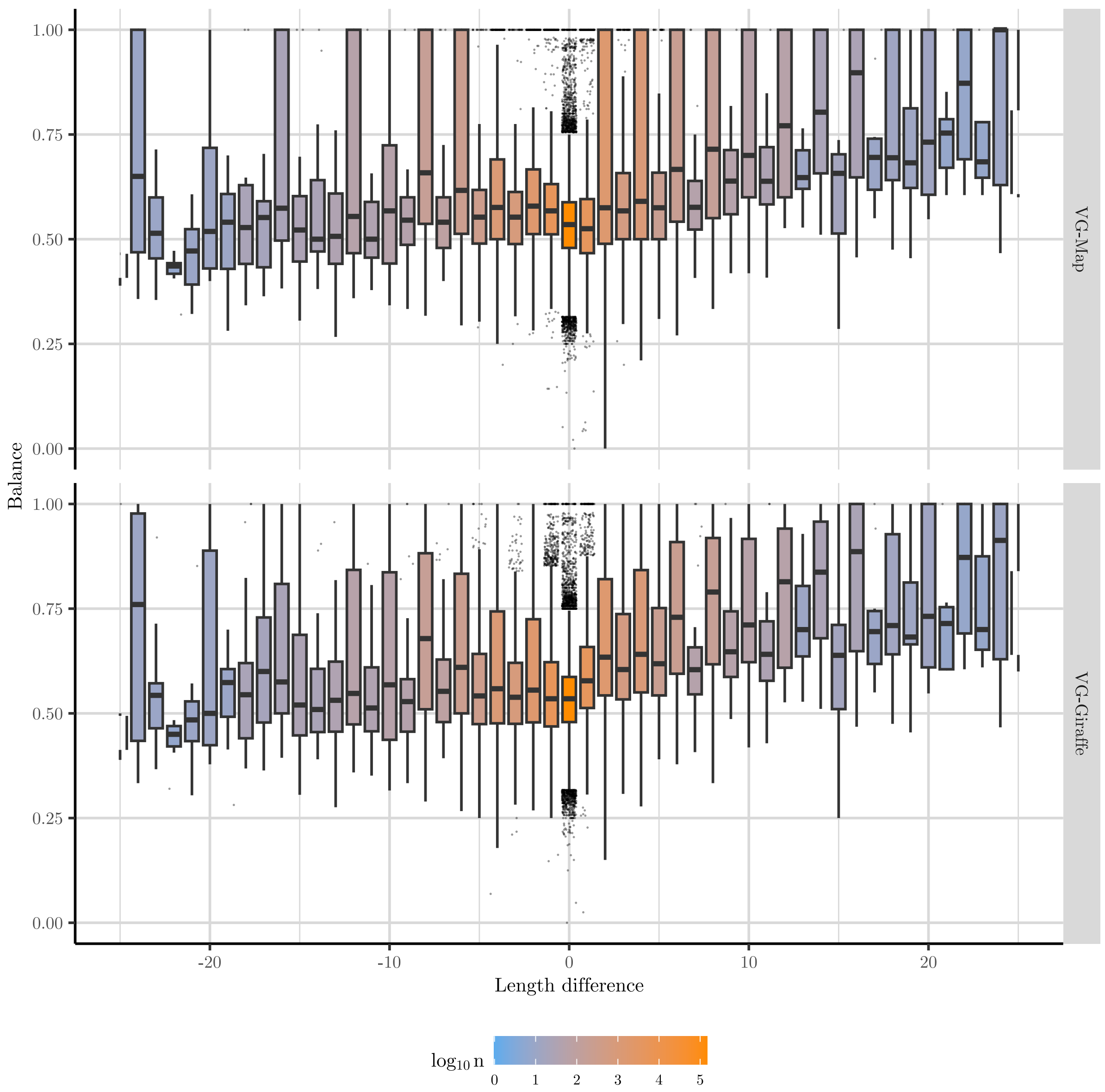

(b) Results for the VG-MAP and Giraffe workflows.

Supplemental Figure 2: Proportions by the difference of the lengths of the alternative allele and the reference allele in the reference bias experiment (continued).

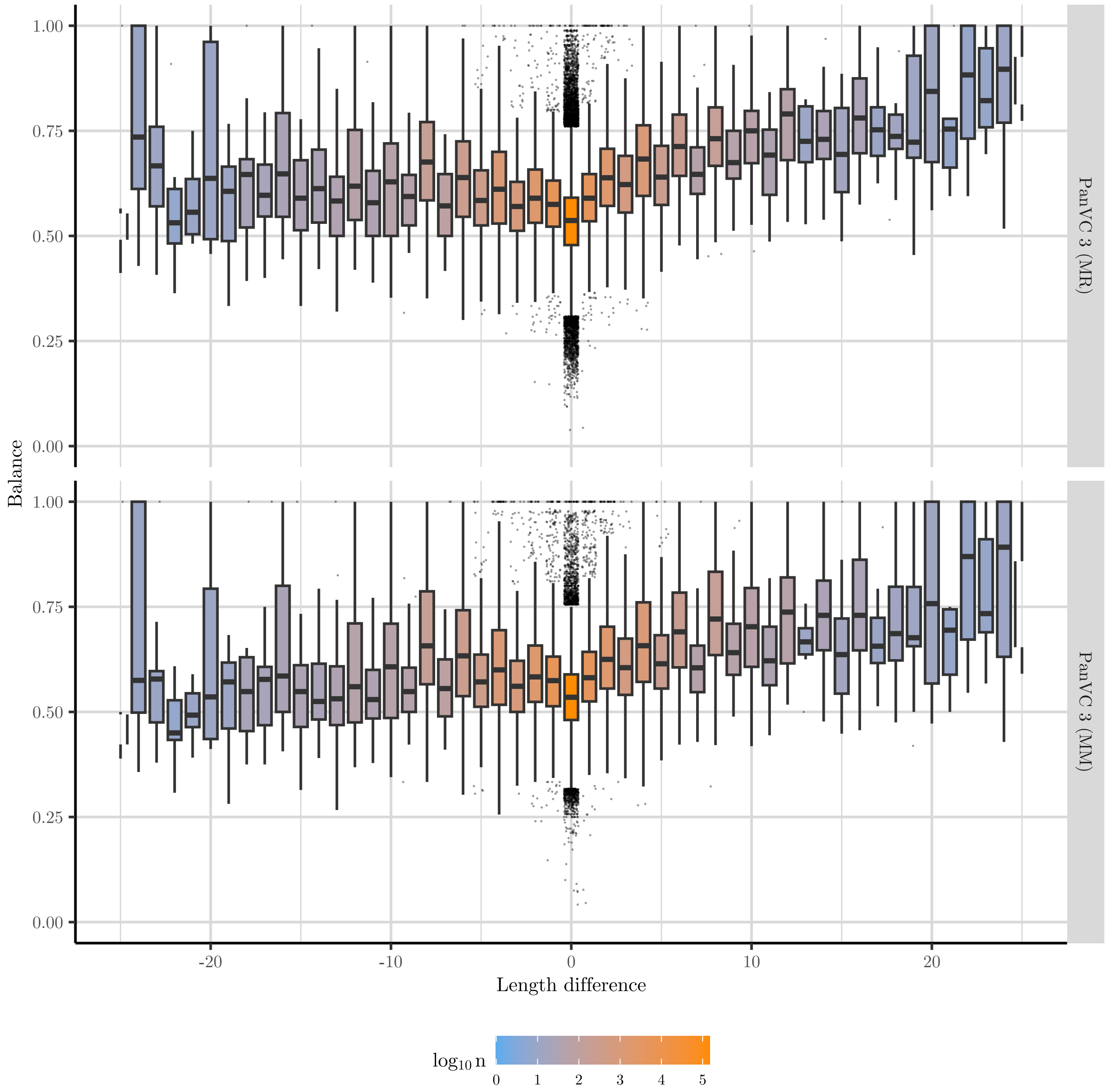

(c) Results for the PanVC 3 (MR) and PanVC 3 (MM) workflows.

Supplemental Figure 2: Proportions by the difference of the lengths of the alternative allele and the reference allele in the reference bias experiment (continued).

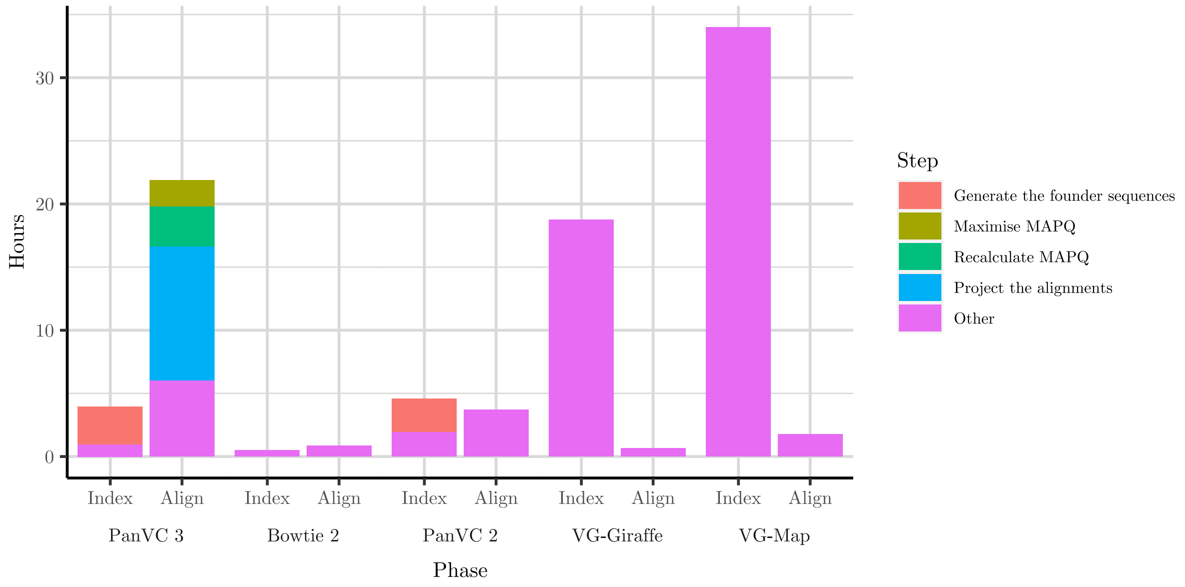

Supplemental Figure 3: Measured wall clock times spent on executing selected workflows in the reference bias experiment. In case of PanVC 2 and 3, some steps are shown separately.

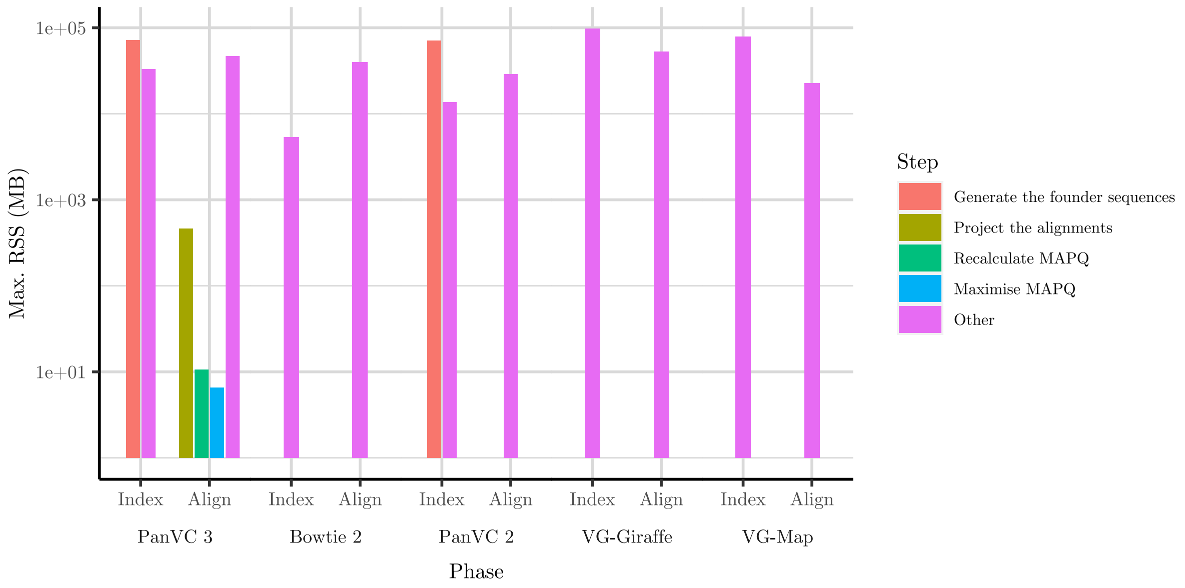

Supplemental Figure 4: Maximum resident set sizes when executing selected workflows in the reference bias experiment. In the case of PanVC 2 and 3, some steps are shown separately.

Supplemental Table 3: Precision and recall as reported by Truvari and the called variant counts in the structural variant calling experiment, considering all regions of chromosome 1 and only variants that passed all filters. MM indicates that the alignments were filtered by maximising the mapping quality while MR indicates that no filtering was done. The rows have been sorted in descending order by precision.

Workflow	PanVC's post-processing	Founder sequences	Distance between subgraphs	Variant caller	Count	Precision	Recall
PanVC 3	MR	15	25	Manta	166	0.8012	0.1396
PanVC 3	MM	8	10	Manta	109	0.7982	0.0913
PanVC 3	MM	3	2	Manta	151	0.7947	0.1259
PanVC 3	MR	8	10	Manta	158	0.7911	0.1312
PanVC 3	MR	26	50	Manta	165	0.7818	0.1354
PanVC 3	MM	26	50	Manta	54	0.7778	0.0441
PanVC 3	MR	3	2	Manta	160	0.7750	0.1301
PanVC 3	MM	15	26	Manta	92	0.7717	0.0745
VG-MAP				Manta	436	0.7225	0.3305
VG-Giraffe				Manta	544	0.6250	0.3568
Bowtie 2				Manta	234	0.5897	0.1448
VG-Giraffe				GRIDSS	0	0.0000	0.0000
VG-MAP				GRIDSS	0	0.0000	0.0000
PanVC 3	MM	8	10	GRIDSS	0	0.0000	0.0000
PanVC 3	MM	26	50	GRIDSS	3886	0.0000	0.0000
Bowtie 2				GRIDSS	4372	0.0000	0.0000
PanVC 3	MR	26	50	GRIDSS	5821	0.0000	0.0000
PanVC 3	MM	15	25	GRIDSS	3878	0.0000	0.0000
PanVC 3	MR	15	25	GRIDSS	5797	0.0000	0.0000
PanVC 3	MM	3	2	GRIDSS	3712	0.0000	0.0000
PanVC 3	MR	3	2	GRIDSS	5529	0.0000	0.0000
PanVC 3	MR	8	10	GRIDSS	5672	0.0000	0.0000

Bowtie 2's default mode in which it reports the best alignment for each read was used. In the workflows which used PanVC 3, Bowtie 2 was asked to search for up to a number of valid alignments equal to the number of generated founder sequences plus one for each read.

In addition to Manta, we tested GRIDSS (Cameron *et al.*, 2021) with all tested aligners. While the variant caller reported several thousands of variants with Bowtie 2 and PanVC 3, the precision and the recall reported by Truvari were zero. It is possible that the variants could not be evaluated by Truvari (e.g. due to breakends).

3.3 Take-One-Out Experiment with a Human Chromosome

We tested the current and the previous version of PanVC with the workflows shown in Supplemental Figure 1. The variant projection algorithm of the previous version of PanVC ("PanVC 2") was improved to detect more cases where comparing the ad hoc reference to the standard reference indicated opposite results to those in the called variants relative to the ad hoc reference. One such situation occurred when a subsequence had been deleted from the ad hoc reference but the called variants suggested that at least one chromosome copy contained the deleted sequence.

Supplemental Table 4: Precision and recall as reported by Truvari and the called variant counts in the structural variant calling experiment, considering confident regions of chromosome 1 and only variants that passed all filters. MM indicates that the alignments were filtered by maximising the mapping quality while MR indicates that no filtering was done. The rows have been sorted in descending order by precision.

Workflow	PanVC's post-processing	Founder sequences	Distance between subgraphs	Variant caller	Count	Precision	Recall
PanVC 3	MM	3	2	Manta	102	0.9706	0.1358
PanVC 3	MM	26	50	Manta	33	0.9697	0.0439
PanVC 3	MM	8	10	Manta	77	0.9610	0.1015
PanVC 3	MR	15	25	Manta	116	0.9569	0.1523
PanVC 3	MR	8	10	Manta	111	0.9550	0.1454
PanVC 3	MR	3	2	Manta	110	0.9545	0.1440
PanVC 3	MM	15	25	Manta	60	0.9500	0.0782
VG-MAP				Manta	271	0.9483	0.3525
PanVC 3	MR	26	50	Manta	116	0.9483	0.1509
VG-Giraffe				Manta	316	0.8987	0.3896
Bowtie 2				Manta	137	0.8467	0.1591
VG-MAP				GRIDSS	0	0.0000	0.0000
VG-Giraffe				GRIDSS	0	0.0000	0.0000
PanVC 3	MM	8	10	GRIDSS	0	0.0000	0.0000
Bowtie 2				GRIDSS	3461	0.0000	0.0000
PanVC 3	MR	26	50	GRIDSS	4118	0.0000	0.0000
PanVC 3	MM	15	25	GRIDSS	2840	0.0000	0.0000
PanVC 3	MR	15	25	GRIDSS	4137	0.0000	0.0000
PanVC 3	MM	26	50	GRIDSS	2812	0.0000	0.0000
PanVC 3	MM	3	2	GRIDSS	2730	0.0000	0.0000
PanVC 3	MR	3	2	GRIDSS	3999	0.0000	0.0000
PanVC 3	MR	8	10	GRIDSS	4047	0.0000	0.0000

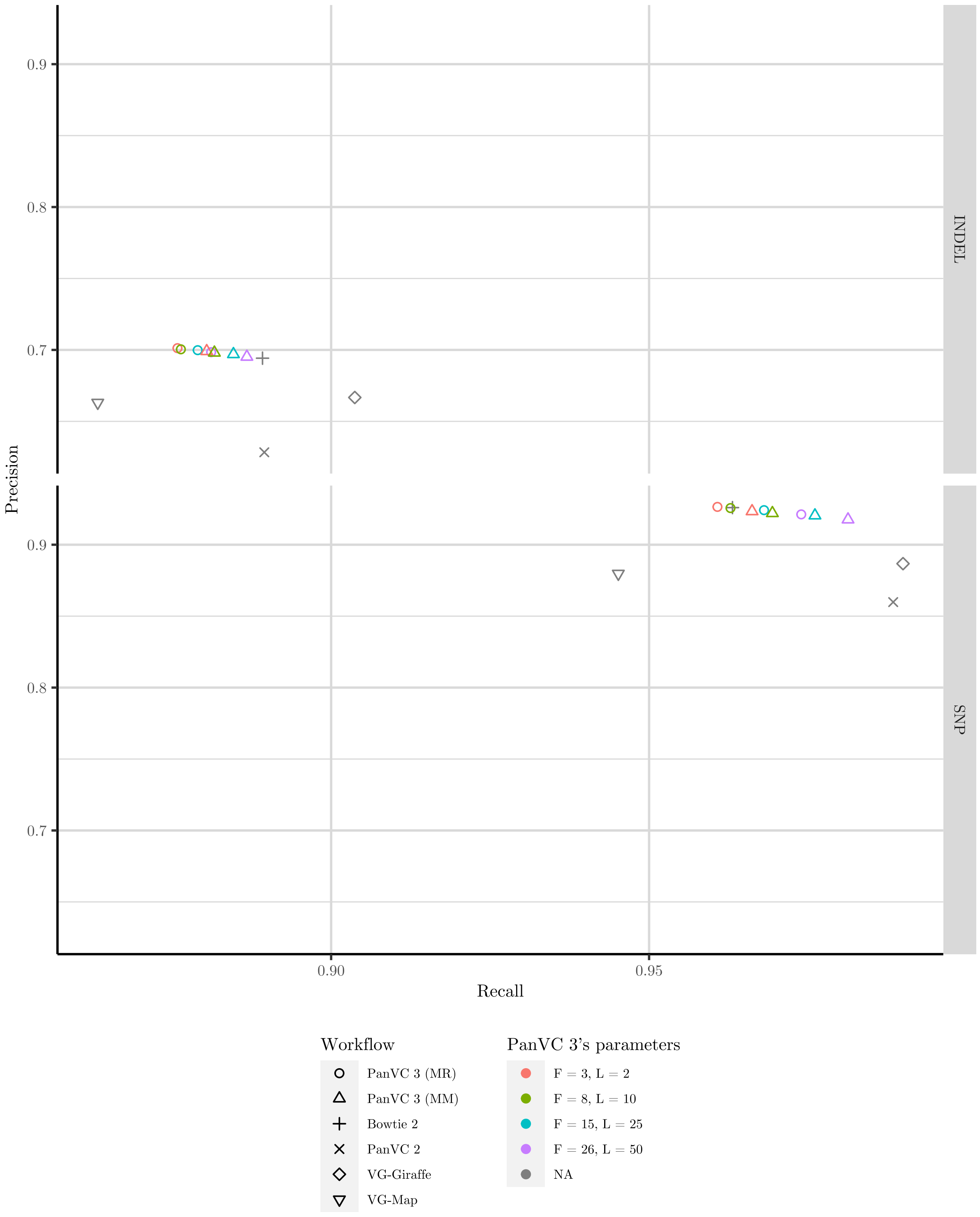

Supplemental Figure 5: Precision and recall as reported by hap.py for short insertions and deletions (top panel) and single nucleotide polymorphisms (bottom panel) in the take-one-out experiment with a human chromosome, considering confident regions of chromosome 1. The point shape corresponds to the workflow and the colour to PanVC 3's parameters. F indicates the founder sequence count including the reference sequence, L is the minimum distance between subgraphs, and MM indicates that the alignments were filtered by maximising the mapping quality while MR indicates that no filtering was done.

4 Further Considerations

While the idea of including additional sequences to the index used as part of read alignment is not new, our method allows the alignment information to be retrieved in an efficient manner regardless of the length or the number of the alternative sequences. Our tools are scalable in the sense that improvements to read alignment could be achieved with a relatively small number of founder sequences. The phases where the alignments are projected and their mapping qualities are recalculated can also be done in parallel per chromosome. Another option in the projection algorithm is to partition the input arbitrarily and process the partitions in parallel. The mapping quality recalculation algorithm needs all the alignments of a given read as its inputs, but with a partitioning that takes the read names into account the same parallelisation method could be applied.

We expected that by adding founder sequences to the index, the measured variant calling results would converge to some value. Instead, we noticed that better results could sometimes be achieved with a smaller number of founder sequences, similar to our experiments with the previous version of PanVC. Our previous hypothesis was that the algorithm for determining the heaviest path used in the previous version could be the cause, but since the new version does not calculate the heaviest path, there seems to be some other reason at least in this case. We did not yet examine the issue but one possible reason is that the sets of founder sequences used as indexing inputs differ in the way the founder segments have been recombined, which then causes the reads to be aligned in another way.

As the experiments indicate that structural variant calling results did improve, a worthwhile further development of our tools is to add means for including structural variants to founder sequences. The resulting multiple sequence alignment will then contain long regions of gaps but it is only needed as input for constructing the co-ordinate conversion data structure.

References

Babenko, M., Gawrychowski, P., Kociumaka, T., and Starikovskaya, T. (2015). Wavelet trees meet suffix trees. In *Proceedings of the 2015 Annual ACM-SIAM Symposium on Discrete Algorithms (SODA)*, pages 572–591.

Cameron, D. L., Baber, J., Shale, C., Valle-Inclan, J. E., Besselink, N., van Hoeck, A., Janssen, R., Cuppen, E., Priestley, P., and Papenfuss, A. T. (2021). Gridss2: comprehensive characterisation of somatic structural variation using single breakend variants and structural variant phasing. *Genome Biology*, **22**(1), 202.

Chen, X., Schulz-Trieglaff, O., Shaw, R., Barnes, B., Schlesinger, F., Källberg, M., Cox, A. J., Kruglyak, S., and Saunders, C. T. (2015). Manta: rapid detection of structural variants and indels for germline and cancer sequencing applications. *Bioinformatics*, **32**(8), 1220–1222.

English, A. C., Menon, V. K., Gibbs, R. A., Metcalf, G. A., and Sedlazeck, F. J. (2022). Truvari: refined structural variant comparison preserves allelic diversity. *Genome Biology*, **23**(1), 271.

Holtgrewe, M. (2010). Mason – a read simulator for second generation sequencing data. *Technical Report FU Berlin*.

Langmead, B. and Salzberg, S. L. (2012). Fast gapped-read alignment with bowtie 2. *Nature methods*, **9**(4), 357–359.

Navarro, G. and Providel, E. (2012). Fast, small, simple rank/select on bitmaps. In R. Klasing, editor, *Experimental Algorithms*, pages 295–306, Berlin, Heidelberg. Springer Berlin Heidelberg.

The 1000 Genomes Project Consortium *et al.* (2015). A global reference for human genetic variation. *Nature*, **526**(7571), 68–74.

The SAM/BAM Format Specification Working Group (2022). Sequence alignment/map format specification. Technical report, Global Alliance for Genomics & Health.

Wagner, J., Olson, N. D., Harris, L., Khan, Z., Farek, J., Mahmoud, M., Stankovic, A., Kovacevic, V., Yoo, B., Miller, N., Rosenfeld, J. A., Ni, B., Zarate, S., Kirsche, M., Aganezov, S., Schatz, M. C., Narzisi, G., Byrska-Bishop, M., Clarke, W., Evani, U. S., Markello, C., Shafin, K., Zhou, X., Sidow, A., Bansal, V., Ebert, P., Marschall, T., Lansdorp, P., Hanlon, V., Mattsson, C.-A., Barrio, A. M., Fiddes, I. T., Xiao, C., Fungtammasan, A., Chin, C.-S., Wenger, A. M., Rowell, W. J., Sedlazeck, F. J., Carroll, A., Salit, M., and Zook, J. M. (2022). Benchmarking challenging small variants with linked and long reads. *Cell Genomics*, **2**(5), 100128.

Zook, J. M., Catoe, D., McDaniel, J., Vang, L., Spies, N., Sidow, A., Weng, Z., Liu, Y., Mason, C. E., Alexander, N., *et al.* (2016). Extensive sequencing of seven human genomes to characterize benchmark reference materials. *Scientific data*, **3**(1), 1–26.

Zook, J. M., Hansen, N. F., Olson, N. D., Chapman, L., Mullikin, J. C., Xiao, C., Sherry, S., Koren, S., Phillippy, A. M., Boutros, P. C., Sahraeian, S. M. E., Huang, V., Rouette, A., Alexander, N., Mason, C. E., Hajirasouliha, I., Ricketts, C., Lee, J., Tearle, R., Fiddes, I. T., Barrio, A. M., Wala, J., Carroll, A., Ghaffari, N., Rodriguez, O. L., Bashir, A., Jackman, S., Farrell, J. J., Wenger, A. M., Alkan, C., Soylev, A., Schatz, M. C., Garg, S., Church, G., Marschall, T., Chen, K., Fan, X., English, A. C., Rosenfeld, J. A., Zhou, W., Mills, R. E., Sage, J. M., Davis, J. R., Kaiser, M. D., Oliver, J. S., Catalano, A. P., Chaisson, M. J. P., Spies, N., Sedlazeck, F. J., and Salit, M. (2020). A robust benchmark for detection of germline large deletions and insertions. *Nature Biotechnology*, **38**(11), 1347–1355.
